# Inhibition of casein kinase 1-epsilon induces cancer-cell-selective, PERIOD2-dependent growth arrest

**DOI:** 10.1186/gb-2008-9-6-r92

**Published:** 2008-06-02

**Authors:** Wan Seok Yang, Brent R Stockwell

**Affiliations:** 1 Department of Biological Sciences, Columbia University, Fairchild Center, Amsterdam Avenue, New York, NY 10027, USA; 2 Department of Chemistry, Columbia University, New York, NY 10027, USA

## Abstract

**Background:**

Kinases are under extensive investigation as targets for drug development. Discovering novel kinases whose inhibition induces cancer-cell-selective lethality would be of value. Recent advances in RNA interference have enabled the realization of this goal.

**Results:**

We screened 5,760 short hairpin RNA clones targeting the human kinome to detect human kinases on which cancer cells are more dependent than normal cells. We employed a two-step screening strategy using human sarcoma cell lines and human fibroblast-derived isogenic cell lines, and found that short hairpin RNAs targeting *CSNK1E*, a clock gene that regulates circadian rhythms, can induce selective growth inhibition in engineered tumor cells. Analysis of gene-expression data revealed that *CSNK1E *is overexpressed in several cancer tissue samples examined compared to non-tumorigenic normal tissue, suggesting a positive role of *CSNK1E *in neogenesis or maintenance. Treatment with IC261, a kinase domain inhibitor of casein kinase 1-epsilon (CK1ε), a protein product of *CSNK1E*, showed a similar degree of cancer-cell-selective growth inhibition. In a search for substrates of CK1ε that mediate IC261-induced growth inhibition, we discovered that knocking down *PER2*, another clock gene involved in circadian rhythm control, rescues IC261-induced growth inhibition.

**Conclusion:**

We identified CK1ε as a potential target for developing anticancer reagents with a high therapeutic index. These data support the hypothesis that circadian clock genes can control the cell cycle and cell survival signaling, and emphasize a central role of CK1ε and PERIOD2 in linking these systems.

## Abstract 2

Casein kinase 1 epsilon is identified as a potential target for developing selective anticancer reagents.

## Background

Cancer can be effectively treated using targeted therapy, as exemplified by Imatinib [1] or Sorafenib [2]. There are increasing efforts to fulfill the promise of targeted therapy, using antibodies, peptides and small molecules that selectively affect cancer cells. In each case, the key is to identify target molecules that play a unique role in tumor cells.

Genes encoding such target molecules can be discovered by either comparative or functional genomic approaches. Comparative approaches analyze cytogenetic data, genomic sequences, mRNA expression profiles or proteomic profiles, and select target genes or proteins based on differential expression or mutation status. For example, high-throughput sequencing of cancer cell genomes identified *BRAF *[3] and *PIK3CA *[4] as frequently mutated genes in multiple human tumors. On the other hand, functional approaches involve perturbing cells with agents, such as cDNAs, small RNAs, or small molecules, and searching for those that induce specific phenotype changes. Subsequent target identification may lead to the discovery of cancer therapeutic targets. Indeed, the *RAS *oncogenes were identified using an expression cloning strategy that searched for human genes that transform the mouse fibroblast cell line NIH3T3 [5].

Among the agents used for functional genomic approaches, small RNAs are increasingly appealing, because RNA-interference (RNAi) mediated by small RNAs enables gene silencing in mammalian cells. RNAi is a naturally occurring phenomenon involved in the silencing of genes, which results in regulation of gene expression or activation of an antiviral defense system [6]. The RNAi pathway involves DICER, which processes double-stranded RNAs into small RNA duplexes (approximately 22 nucleotides). One strand of the small RNA duplex is incorporated into an effector complex known as the RNA-induced silencing complex (RISC) and acts as a guide molecule in translational repression or mRNA cleavage, depending on the degree of base-pair match with the target mRNA [7].

The conserved RNAi pathway is also activated by experimentally designed double-stranded RNAs or short hairpin RNAs (shRNAs), which make it possible to knock down genes of interest in mammalian cells. Consequently, RNAi libraries targeting large numbers of mRNAs have been generated and used for conducting high-throughput, loss-of-function screens in tissue culture systems. For example, RNAi libraries were used to identify novel tumor suppressors [8,9], regulators of cell death and survival [10], and novel components of p53 signaling [11]. Moreover, RNAi libraries were used for understanding the mechanisms of action of novel compounds [12], for characterizing determinants of sensitivity to clinically used drugs [13], and for identifying novel targets for anti-cancer therapy, using a pair of isogenic cell lines [14].

Isogenic cell lines are useful for discovering therapeutic agents and probing the biology of transformation. They may consist of cancer cells at different stages of malignancy, or a specific cancer gene can be deleted to create an isogenic cell line counterpart. Another approach is to isolate primary cells and induce transformation by sequential addition of oncogenic elements. This system provides a series of genetically defined cell lines, and thereby allows for identification of tumor-cell-selective, or even genotype-selective, lethal agents. The successful use of such a system has been described for identification of small molecules with potentially high therapeutic indices [15].

Here we utilized an RNAi library consisting of shRNAs targeting human kinases to find kinases whose inactivation induces tumor-cell-selective lethality or growth arrest. The initial screening was conducted in two sarcoma cell lines; then, a series of isogenic cell lines derived from primary fibroblasts were used for selecting tumor-cell-specific cytotoxic shRNAs. We report that knocking down *CSNK1E*, a clock gene encoding casein kinase 1-epsilon (CK1ε), induces tumor-cell-selective cytotoxicity. Subsequent validation experiments showed that tumor cells depend more on the kinase activity of CK1ε than normal cells do. The use of a kinase inhibitor specific to CK1ε revealed that another clock protein, PERIOD2, is a key substrate of CK1ε and modulates tumor cell growth.

## Results and discussion

**Figure 1:**
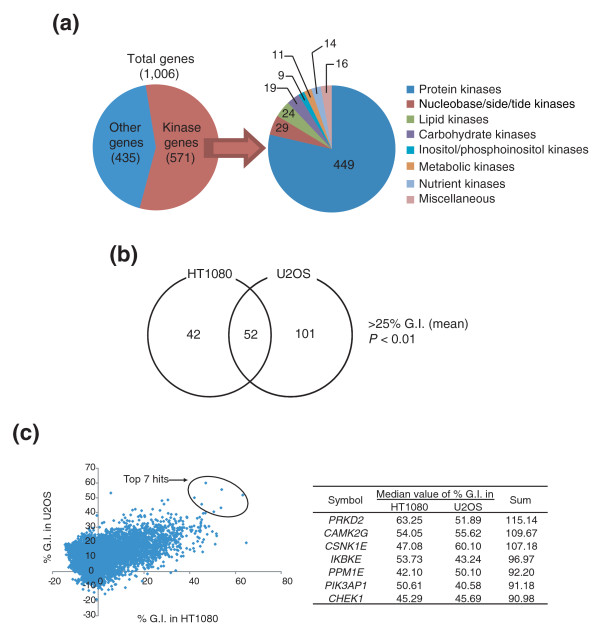
Lentiviral RNAi library screen of human kinases identifies regulators of cancer cell growth. **(a) **Target genes covered by the shRNA library were classified according to gene function using Gene Ontology groups [33]. **(b) **Two human sarcoma cell lines, HT1080 and U-2-OS cells, were infected with lentiviruses containing shRNAs targeting human kinases in 384-well format. Genes whose knockdown inhibits growth of either cell line by more than 25% compared to control with statistical significance (*P *< 0.01) were considered as hits. This diagram shows the number of hits specific to each cell line and common to both cell lines. **(c) **Median value of percent growth inhibition (%GI) from triplicate results in each cell line. The top seven hits were selected based on the summed value of %GI in both cell lines.

Our RNAi library was made of lentivirus solutions in 384-well plates. Each well contains lentiviruses harboring expression plasmids encoding a single shRNA that is designed to target a single mRNA. The arrayed library targets 1,006 human genes, including 571 kinases; most of them are protein kinases, while other kinases acting on nucleic acids, lipids, and carbohydrates are included (Figure 1a).

We began the screen with two different sarcoma cell lines, with the goal of pre-selecting shRNAs that are lethal to these tumor-derived cell lines. We infected U-2-OS, osteosarcoma-derived cells, and HT1080, fibrosarcoma-derived cells, in triplicate and incubated them for three days. This allows time for the shRNAs to be expressed, to bind to their target mRNAs, and cause a reduction in expression of the encoded protein, as the protein is turned over. Percent growth inhibition was determined by adding alamar blue to the culture, and by measuring fluorescence.

A number of shRNA clones displayed growth inhibitory effects (Figure 1b,c and Additional data file 1). Statistical analysis of primary screening data revealed 195 genes whose knockdown inhibited growth of either cell lines more than 25% (*P *< 0.01; Figure 1b and Additional data file 1). Fifty-two genes affected cell growth in both cell lines, while other genes had specific effects on each cell line, which may reflect the different tissue origin of these two sarcomas. Some of the hit genes in common between the two cell lines are well-known regulators of the cell cycle or cell survival, but there were nine genes whose functions have not been described (Additional data file 1). We were most interested in genes whose functions are most critical to the survival of these two cancer cell lines. Accordingly, we calculated the sum of the percent growth inhibition in each cell line and selected seven shRNAs whose summed values were >90% (Figure 1c). Six out of these seven genes were among the 52 common hits in Figure 1b. One gene, *PPM1E*, was not statistically significant and, as expected, shRNAs targeting *PPM1E *were not active in our follow-up analysis (data not shown).

Reducing expression of these six target genes causes growth arrest or cell death in two different sarcoma-derived cancer cell lines; however, we were concerned that knockdown of these six genes may affect normal cells to the same degree. To identify shRNA clones that have cancer cell selectivity, we created fresh batches of lentivirus harboring the six shRNA clones and retested them in a pair of nearly isogenic cell lines, BJ-TERT and BJ-TERT/LT/ST/RAS^V12^. Both cell lines were derived from primary human BJ foreskin fibroblasts [16]. These BJ primary cells were engineered successively to express the catalytic subunit of human telomerase (hTERT), the SV40 large T and small T oncoproteins (LT and ST), and an oncogenic allele of *HRAS *(HRAS^G12V^). We refer to these cells lines as BJ-TERT, BJ-TERT/LT/ST, and BJ-TERT/LT/ST/RAS^V12^. Only the BJ-TERT/LT/ST/RAS^V12 ^cells form tumors in nude mice. Therefore, testing of shRNAs in BJ-TERT and BJ-TERT/LT/ST/RAS^V12 ^should enable one to identify genes with a function that is essential in tumor cells, but not normal cells. We measured trypan blue exclusion to evaluate the cytotoxic or growth inhibitory effect of these shRNA clones on normal cells and their isogenic engineered tumor cell counterparts.

**Figure 2:**
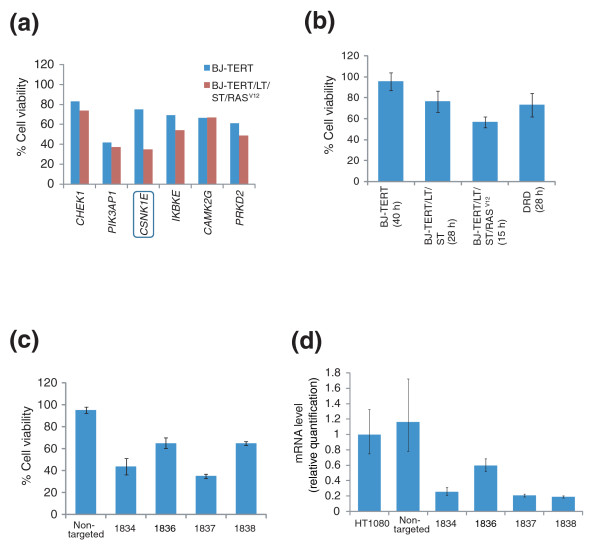
*CSNK1E *is a target for developing anti-cancer drugs with a potentially high therapeutic index. **(a) **Retesting of six hit shRNA clones in isogenic BJ-derived cell lines. Knocking down *CSNK1E *induced cancer-cell-specific growth inhibition, whereas knocking down other survival genes did not display differential activity in the two cell lines. The graph is representative of multiple experiments. **(b) **The activity of *shCSNK1E *was examined in four isogenic BJ-derived cell lines. The growth inhibitory effect of *shCSNK1E *was proportional to the cell proliferation rate. The doubling time of each cell line is shown in parentheses. **(c) **Inhibition of HT1080 cell growth by independent shRNA clones that bind to different regions of *CSNK1E *mRNA. **(d) **The knockdown efficiency of each shRNA clone targeting *CSNK1E *as assessed by quantitative PCR analysis. Error bars in (b-d) indicate one standard deviation of triplicate data.

Out of these six shRNA clones, five did not show differential activity in the two cell lines; however, the shRNA targeting *CSNK1E *(hereafter, *shCSNK1E*) had a tumorigenic-cell-line-specific activity (Figure 2a). The activity of *shCSNK1E *was further tested in four different BJ-derived cell lines, namely, BJ-TERT, BJ-TERT/LT/ST, BJ-TERT/LT/ST/RAS^V12^, and DRD cells. DRD cells were engineered to express hTERT, ST, HRAS^G12V^, dominant negative p53, and constitutively active cyclin-dependent kinase (CDK)4/cyclin D, which inactivates the RB protein [17]. The p53DD/CDK4/cyclin D1 combinations substitute for LT. DRD cells are tumorigenic in nude mice, which is expected from the fact that they are also derived from BJ primary cells and the effects of mutations in both cell lines should be similar. The growth inhibitory potential of *shCSNK1E *increased as the cell doubling time decreased, suggesting that the activity is proliferation-rate dependent rather than genotype dependent (Figure 2b).

Theoretically, the length of shRNA involved in base paring with the target mRNA is long enough to ensure specificity of the shRNA clone. However, mismatches between a shRNA and target mRNAs are tolerable; RISC is able to suppress expression of off-target mRNAs whose sequences do not perfectly complement the guide strand of the shRNA [7]. In order to confirm our hypothesis that knocking down expression of *CSNK1E *is responsible for the observed growth inhibition, we tested multiple shRNA clones targeting the *CSNK1E *gene; each shRNA clone binds to different regions of the *CSNK1E *mRNA. If more than a single shRNA clone induces growth inhibition, *CSNK1E *is likely to be the relevant target, because the probability of a common off-target effect of multiple shRNA clones with unrelated sequences is low. We found that four shRNAs targeting *CSNK1E *induced strong growth inhibition in HT1080 cells (Figure 2c). The level of *CSNK1E *mRNA decreased upon expression of these shRNAs, as assessed by real-time quantitative PCR analysis (Figure 2d). Note that one of these shRNAs, clone 1838, did not display stronger growth inhibition effects even though the mRNA level was decreased significantly. This is likely to reflect an off-target effect of this particular shRNA.

**Figure 3:**
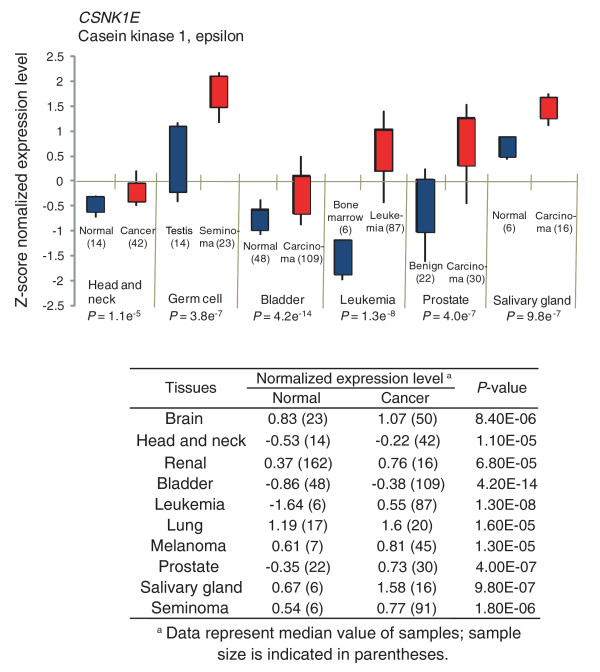
Gene expression studies comparing normal and cancer tissues were analyzed for *CSNK1E *using Oncomine [21]. *CSNK1E *was found to be over-expressed in cancer samples over normal samples regardless of tissue origin. The graph shows representative results of *CSNK1E *gene expression analysis from six human tissues. The number of samples in each study is provided in parentheses. The y-axis units are based on z-score normalization and the *P*-value of each set is shown at the bottom of the graph. The upper and lower bands of the box represent the 75th and 25th percentiles, respectively; the upper and lower error bars represent the 90th and 10th percentiles, respectively. The table shows normalized expression levels of *CSNK1E *in normal and cancer samples from ten human tissues.

The *CSNK1E *gene encodes the CK1ε protein, whose main function is to regulate the circadian rhythm by phosphorylating other clock gene products [18]. The role of CK1ε in cancer has been speculated upon, because CK1ε was shown to phosphorylate key proteins in cancer signaling pathways, such as p53 [19] and β-catenin [20]. However, the significance of these phosphorylation events in carcinogenesis is not known, and the possibility of using CK1ε as a pharmacological target for cancer treatment has not been considered. Therefore, we analyzed the expression level of *CSNK1E *in human tumor samples to obtain support for its involvement in human cancer. Some genes that are specifically required for tumor maintenance are overexpressed in cancer cells over normal cells. We analyzed the gene-expression database Oncomine for differential expression patterns in normal versus tumor in different tissue types [21]. The Oncomine database contained microarray expression data for *CSNK1E *from ten different tissues, including brain, head and neck, renal, bladder, leukemia, lung, melanoma, prostate, salivary gland, and seminoma. Interestingly, all tumor tissues in the database showed upregulated *CSNK1E *expression compared to normal tissues, suggesting a positive role of CK1ε in cancer maintenance or neogenesis (Figure 3).

**Figure 4:**
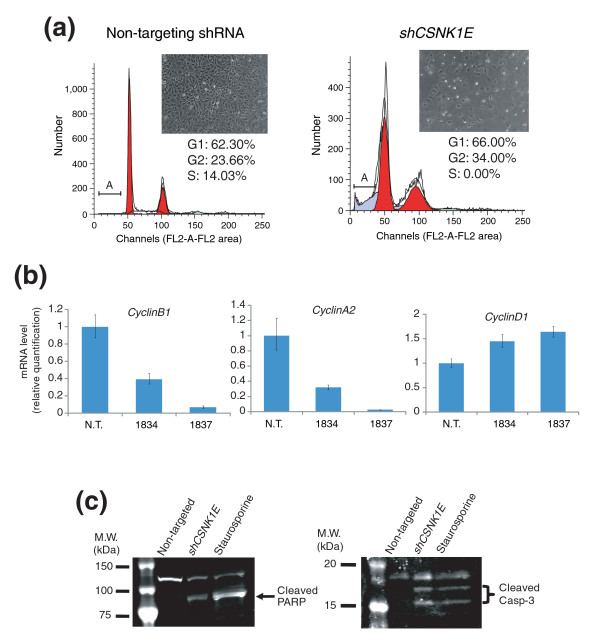
Knocking down *CSNK1E *induces G2/M cell cycle arrest and caspase-mediated apoptosis. **(a) **Two days after non-targeting shRNA or *shCSNK1E *treatment, HT1080 cells were fixed in methanol and stained with propidium iodide (Materials and methods). Flow cytometry of cells was performed on a FACSCalibur; calculation of cell cycle stages was performed using the cell cycle analysis program Modifit LT. Red area shows cell population in G1 or G2 cell cycle phase, while gray area shows dying cells. Label 'A' denotes apoptotic cell population. Insets show photographs of HT1080 cells treated with non-targeting shRNA or *shCSNK1E*. **(b) **Knocking down *CSNK1E *down-regulates *CyclinB1 *and *CyclinA2*. Cellular RNAs were prepared from HT1080 cells infected with either non-targeting shRNA (N.T.) or two different *CSNK1E*-targeting shRNAs (1834, 1837), and real-time PCR was performed with each gene-specific primer set. The expression levels of *CyclinB1*, *CyclinA2 *and *CyclinD1 *were first normalized to the level of an endogenous control (*RPLPO*), and then the relative expression level of each gene among the three cell lines was expressed as a ratio of transcripts in a cell line to those in non-targeted shRNA treated cells. Error bars indicate one standard deviation of triplicate data. **(c) **Knocking down *CSNK1E *induces caspase activation. Whole cell lysates from HT1080 cells infected with either non-targeting shRNA or *shCSNK1E *and cells treated with staurosporine were prepared. The cleavage of PARP1 or caspase-3 (Casp-3) in each sample was examined by western blotting using antibodies against PARP1 and cleaved caspase-3.

The proliferation-rate-dependent action of *shCSNK1E *(Figure 2b) raises the possibility that shRNA treatment induces cell cycle arrest; thus, fast growing cells have greater growth inhibition. To test this hypothesis, we stained the DNA of shRNA-treated cells with propidium-iodide and analyzed the cell cycle distribution by flow cytometry. The cell cycle distribution profile indicates that, after expression of *shCSNK1E*, HT1080 cells were arrested in the second gap (G2) phase of the cell cycle, with a concomitant increase in the population of cells harboring less than the normal diploid DNA content (that is, sub-G1), implying apoptosis had occurred (Figure 4a).

The cell cycle is primarily regulated by the activity of cyclins and CDKs. Among CDK/cyclin complexes, CDK1-cyclin A promotes the transition from G2 to mitosis (M), while CDK1-cyclin B governs maturation of M phase [22]. We examined whether *shCSNK1E *treatment affected expression of cyclin A2 and cyclin B1 in HT1080 cells. Real-time PCR analysis revealed that *shCSNK1E *decreased mRNA levels of cyclin B1 and cyclin A2 (Figure 4b). In contrast, mRNA levels of cyclin D1, whose function is important for the G1 to S transition, were slightly increased (Figure 4b). These data are consistent with the cell cycle distribution pattern after *shCSNK1E *treatment observed by flow cytometry; knocking down *CSNK1E *expression caused down-regulation of cyclin B1 and cyclin A2, which results in cell cycle arrest at the G2/M phase.

In addition to G2/M phase cell cycle arrest, *shCSNK1E *treatment induced apoptotic cell death, as evidenced by the appearance of small, fragmented cells and a sub-G1 population (Figure 4a). To confirm the apoptotic phenotype of *shCSNK1E*-treated cells, we examined cleavage of poly(ADP-ribose)polymerase-1 (PARP1), which is cleaved by caspases during apoptosis. Western blot analysis with antibodies specific to PARP1 showed that cells treated with a non-targeting shRNA contained only full length PARP1, whereas those treated with *shCSNK1E *or staurosporine, a known inducer of caspase-dependent apoptosis, contained a diagnostic PARP1 fragment, indicating that apoptotic caspases were activated by these treatments (Figure 4c). Activation of apoptotic caspases was further confirmed by western blot, which detected the active form of caspase-3 only in *shCSNK1E *or staurosporine-treated samples (Figure 4c). Thus, *shCSNK1E *induces caspase-mediated apoptosis in sensitive cancer cells.

**Figure 5:**
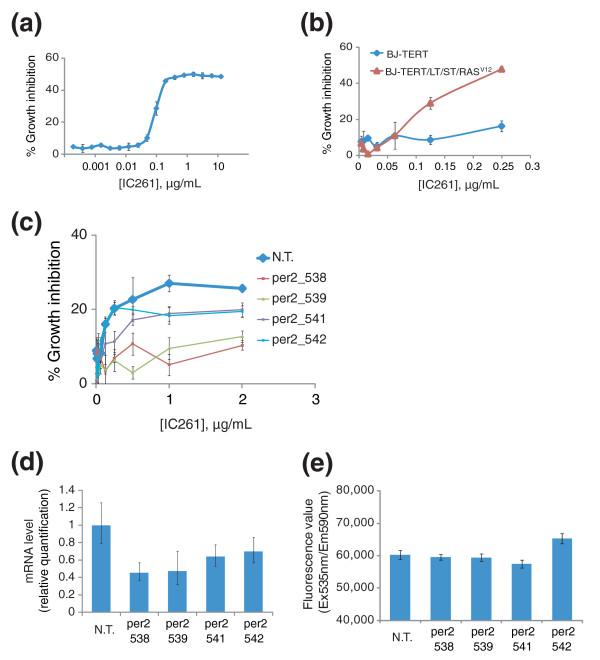
PERIOD2 is a key substrate of CK1ε that mediates IC261-induced growth inhibition. **(a) **IC261, a kinase inhibitor of CK1ε, induces growth inhibition in HT1080 cells. **(b) **IC261 treatment in BJ-derived cell lines showed a similar degree of cancer cell selective growth inhibition as *shCSNK1E *treatment. **(c) **Knocking down *PER2 *in HT1080 cells rescues growth inhibition induced by IC261. HT1080 cells were infected with indicated lentiviruses containing different shRNA clones targeting *PER2 *(per2_538, per2_539, per2_541, or per2_542). After two days of infection, cells were treated with the indicated concentration of IC261 and percent growth inhibition was determined using alamar blue. Values in (a-c) represent the mean ± standard deviation of triplicate data. **(d) **Cellular RNAs were prepared from the same set of virus infected cells in (c), and real-time PCR was performed with a *PER2*-specific primer set to monitor the efficiency of knock down by shRNA clones. **(e) **Proliferation rate of HT1080 cells infected with the same set of viruses as in (c) was determined using alamar blue assay. Error bars in (d,e) indicate one standard deviation of triplicate data. N.T., non-targeting shRNA clone.

These results suggest that chemotherapeutic reagents targeting CK1ε may induce growth arrest and apoptosis with some degree of cancer cell selectivity. To test this hypothesis, we examined the effect of IC261, a kinase inhibitor of CK1ε, in cell culture. IC261 was reported to selectively inhibit casein kinase 1 compared to other protein kinases, by an ATP-competitive mechanism. Moreover, it showed an order of magnitude greater selectivity for CK1δ and CK1ε over other casein kinase 1 isoforms [23]. Treatment with IC261 started to inhibit the growth of HT1080 cells at submicromolar concentrations (Figure 5a). When we tested IC261 in BJ-TERT and BJ-TERT/LT/ST/RAS^V12 ^cells, the sensitivity of BJ-TERT/LT/ST/RAS^V12 ^cells was greater than that of BJ-TERT cells, which was consistent with the results obtained with *shCSNK1E *(Figures 5b and 2a). These data suggest that inhibition of the kinase activity of CK1ε is crucial for the observed growth arrest and apoptosis, as opposed to other functions of this protein, such as those mediated by protein-protein interactions. Moreover, as shRNAs targeting CK1δ were not effective in suppressing cell growth during primary screening, the cancer-cell-selective activity of IC261 can likely be attributed to its inhibition of CK1ε (Additional data file 1).

CK1ε is known to control the circadian rhythm by phosphorylating clock proteins, such as PERIOD and CRYPTOCHROME [24]. These clock proteins are also reported to regulate the cell cycle, suggesting they have a role in linking the circadian system and the cell cycle machinery [25,26]. Mammalian cells have three isoforms of PERIOD proteins and two isoforms of CRYPTOCHROME proteins, which are encoded by *PER1*, *PER2*, *PER3*, *CRY1 *and *CRY2 *genes, respectively. In order to define the role of each isoform in CK1ε-mediated growth regulation, we conducted counter-screening with shRNAs targeting these genes to identify suppressors of IC261-induced growth inhibition in HT1080 cells. Knocking down expression of *PER1*, *PER3*, *CRY1*, or *CRY2 *did not affect growth inhibition by IC261 (Additional data file 2). However, four different shRNA clones targeting *PER2 *suppressed IC261-induced growth inhibition, implying that PERIOD2 is the most crucial substrate of CK1ε in controlling cell proliferation (Figure 5c,d). Note that the maximum growth inhibition by IC261 in Figure 5c is smaller than that in Figure 5a, though they have similar EC50 values of 0.1 μg/ml. This is because cells have been growing for three days before being treated with IC261 in order to express shRNAs targeting *PER2*, whereas in Figure 5a, IC261 was added to culture at the time of cell seeding.

As we showed that the proliferation rate of target cells is an important determinant of growth inhibition by *CSNK1E *knockdown (Figure 2b), we measured the proliferation rate of HT1080 cells upon *PER2 *knockdown using the alamar blue assay (Figure 5e). None of the shRNA clones targeting *PER2 *changed the proliferation rate of HT1080 cells, indicating that the protective effect of *PER2 *knockdown on IC261-induced growth inhibition is not caused by slowing cell growth. It has been shown that a major function of CK1ε in the circadian rhythm is to phosphorylate PERIOD2, which drives proteosome-mediated degradation of PERIOD2 [27]. In several independent reports, overexpression of PERIOD2 has been shown to exert anti-tumor effects in both cell culture and mouse models [26,28,29]. Therefore, treatment with IC261 is likely to stabilize PERIOD2, which activates the PERIOD2-mediated tumor suppression pathway.

Here, we report the identification of CK1ε as a potential target for developing anticancer reagents. The mammalian CK1 family consists of at least seven isoforms (α, β, γ1, γ2, γ3, δ and ε), as well as additional splice variants [18]. They share highly conserved kinase domains, but differ significantly in the length and primary structure of their amino- and carboxy-terminal non-catalytic domains, implying that each isoform may play a specific role in regulating biological processes [18]. Defining isoform-specific functions will aid us in developing agents with enhanced specificity and reduced off-target effects. As the specificity of RNAi agents is potentially high, it allows us to differentiate among these isoforms, which is challenging for some chemical inhibitors.

In our screening, knocking down other isoforms of CK1 was not effective at inducing growth arrest, implying that CK1ε has a unique function in promoting the integrity and proliferation of tumor cells. The nature of the signaling pathway that CK1ε uses to control cell growth remains elusive, but several lines of evidence support a positive role of this kinase in oncogenesis. First, in our gene expression analysis, cancer cells have a high level of CK1ε compared to normal cells, regardless of the tissue origin, implying that a high level of CK1ε causes a growth or survival advantage during tumorigenesis (Figure 3). Second, in a recent report, enforced expression of myristoylated-CK1ε, but not other isoforms, induced colony formation in soft-agar-growing engineered human epithelial cells [30]. Third, deletion of PERIOD2 in mice caused increased tumor development upon gamma-radiation, suggesting a tumor suppressive role of PERIOD2 [26]. CK1ε is a major kinase that phosphorylates and degrades the PERIOD2 protein through the proteasome [31]; therefore, it is likely that CK1ε exerts its oncogenic effect by inhibiting the tumor suppressive function of PERIOD2. In accordance with this model, we showed that knocking down *PER2 *abrogated the growth inhibitory effect of IC261, a kinase inhibitor of CK1ε (Figure 5c).

## Conclusion

RNAi libraries and isogenic cell lines make it possible to identify target genes and proteins for cancer therapeutic development. We found that *CSNK1E *is one such target gene upon which cancer cells depend more than normal cells. As kinase inhibitors of CK1ε displayed the same phenotype as shRNA treatments, efforts to develop kinase inhibitors of CK1ε with enhanced potency and selectivity would be valuable. Future work involving the screening of larger shRNA libraries might reveal additional potential drug targets.

## Materials and methods

### Cell lines

The human fibrosarcoma cell line HT1080 was maintained in Dulbecco's modified Eagle's medium (DMEM) supplemented with non-essential amino acids and 10% calf serum. The human osteosarcoma cell line U-2-OS was grown in McCoy's 5A medium supplemented with 10% calf serum. BJ-fibroblast-derived cell lines were grown in a 4:1 mixture of DMEM to M199 supplemented with 15% heat-inactivated fetal bovine serum. Penicillin and streptomycin were used as antibiotics in all media. All cells were incubated in a tissue culture incubator at 37°C in a humidified incubator containing 5% CO_2_.

### Lentiviral shRNA library

We used a library targeting human kinases for our screening that was generated by The RNAi Consortium [32]. Our shRNA library consists of lentivirus solutions in 384-deep-well polypropylene plates (Greiner, Monroe, NC, USA, catalog number 781270). The library targets 1,006 human genes, including kinases, those similar to kinases and some ancillary proteins. The lentivirus in each well contains an expression cassette (pLKO.1) encoding a single shRNA clone. On average, the library contains five different shRNA clones targeting each gene and has a typical virus titer range from 10^7^-10^8 ^IU (infection unit)/ml. We refer to these plates as virus mother plates.

### Primary screening

Assay plates were prepared by seeding 400 U-2-OS or HT1080 cells per well in 40 μl of growth media in black, clear-bottom, 384-well plates (Corning Inc., Corning, NY, USA, catalog number 3712). The next day, 40 μl of virus daughter plates were prepared by transferring 2 μl of virus stock solution from virus mother plates and 4 μl of 10× polybrene solution to 34 μl of cell growth media in 384-well polypropylene plates (Greiner, catalog number 781280). Whole growth media in the assay plates were replaced with 40 μl of virus/polybrene/media mixture from the virus daughter plates. Then, virus infection was carried out by centrifuging the assay plates for 1.5 h at 2,250 rpm, 37°C and the assay plates were returned to a tissue culture incubator. Three days later, alamar blue was added to the assay plates. All liquid handling was carried out using a Biomek FX AP384 module (Beckman Coulter, Fullerton, CA, USA). Cell viability was measured using alamar blue (Invitrogen, Carlsbad, CA, USA, catalog number DAL1100); subsequently, percent growth inhibition (%GI) was calculated from the following formula using fluorescence intensity values:

%GI = 100 × (1 - (X - N)/(P - N))

where X is values from cells infected with shRNAs, N is the values from media only, and P is the values from cells grown without shRNAs.

All experiments were performed in triplicate and median percent growth inhibition value was taken for selecting final hits to be analyzed.

### Follow-up analysis of hit shRNA clones

#### Virus production

We used lentiviral plasmids encoding shRNAs targeting *CSNK1E *(catalog number SHGLY-NM_001894), *PER1 *(catalog number SHGLY-NM_002616), *PER2 *(catalog number SHGLY-NM_003894), *PER3 *(catalog number SHGLY-NM_016831), *CRY1 *(catalog number SHGLY-NM_004075), or *CRY2 *(catalog number SHGLY-NM_021117). All shRNA clones were obtained from Sigma's MISSION^® ^shRNA collection (Sigma, St. Louis, MO, USA). Plasmid DNA was purified using a HiSpeed Plasmid Midi kit (Qiagen, Valencia, CA, USA, catalog number 12643). On day one, 2 × 10^6 ^293T cells were seeded in 10 cm tissue culture dishes; on day two, 2.8 μg of shRNA-plasmid construct and 2.5 μg of pDelta8.9 and 0.28 μg of pVSV-G helper plasmids were co-transfected into the 293T cells using FuGENE^® ^6 Transfection Reagent (Roche, Indianapolis, IN, USA, catalog number 11-814-443-001); on day three, the medium was replaced with 7.5 ml of viral collection media (VCM) that consists of DMEM supplemented with penicillin and streptomycin (pen/strep), and 30% Hyclone iFCS (Hyclone, Logan, UT, USA, catalog number 83007-198); on day four, in the morning, the supernatant containing virus was harvested to empty 50 ml conical tubes and 7.5 ml of fresh VCM was added back to virus producing 293T cell monolayer. We harvested and replaced the VCM again in the evening; on day five, in the morning, we harvested the supernatant and bleached the 293T cell culture. The collected virus supernatant was filtered through a 0.45 μm syringe filter (Nalgene, Rochester, NY, USA, catalog number 190-9945), aliquoted in 2 ml to the cryovials, and stored at -80°C freezer until time of use.

#### Virus infection

We seeded 200,000 target cells on 10 cm tissue culture dishes and the culture was incubated at 37°C in a CO_2 _incubator for 24 h. The next day, frozen stocks of virus solution were thawed at 37°C for a couple of minutes and polybrene (Sigma, catalog number H9268) was added at a final concentration of 8 μg/ml. Culture media was replaced with virus/polybrene mix and the culture dish was incubated for 2 h with rocking every 30 minutes. After 2 h, 10 ml of growth media was added to culture dish and the culture was incubated further for 2 days before harvesting or treatment of compounds.

#### Retesting shRNA clones in four BJ cell lines

BJ-TERT or BJ-TERT/LT/ST/RAS^V12 ^cells were seeded and infected with lentivirus as described above. After 60 h, infected cells were released with trypsin/EDTA and harvested in 4 ml BJ growth medium. Aliquots of the cell suspension were used for determining cell viability by trypan blue assay. A hit shRNA clone that displayed differential activity between BJ-TERT and BJ-TERT/LT/ST/RAS^V12 ^cells was further tested in BJ-TERT, BJ-TERT/LT/ST, BJ-TERT/LT/ST/RAS^V12^, or DRD cells using the same method. Trypan blue staining, taking 100 images of samples, and analysis of the images were carried out automatically by Vi-Cell (Beckman Coulter).

#### Monitoring drug sensitivity

On the day of the experiment, empty 384-deep-well polypropylene plates (Greiner, catalog number 781270) were filled with 50 μl growth media except for columns 5 and 13, where 100 μl of IC261 solution (100 μg/ml in growth media) was transferred. IC261 is a kinase inhibitor of CK1ε and was purchased from Calbiochem, San Diego, CA, USA (catalog number 400090). After IC261 solution transfer, 2-fold dilution series across columns 5-12 and columns 13-20 were done by transferring 50 μl of compound solution to the next column successively (8-point dilution series) with mixing. We named this plate '10× IC261 plate'. Assay plates were prepared by seeding 1,500 shRNA-infected HT1080 cells per well in 36 μl of growth media to black, clear bottom 384-well plates. Cells in the assay plates were treated with IC261 in a 2-fold dilution series by transferring 4 μl solution from a 10× IC261 plate. Assay plates were returned to the culture incubator and maintained for 24 h before adding alamar blue. Percent growth inhibition was calculated using fluorescence intensity values.

#### Alamar blue assay

After 24 or 48 h of compound treatment, 10 μl of 50% alamar blue solution in growth medium was transferred to the assay plates, which resulted in 10% final concentration alamar blue. Plates were incubated further for 16 h to allow reduction of alamar blue, which results in the generation of red fluorescence. The fluorescence intensity was determined using a Victor 3 plate reader (Perkin Elmer, Waltham, MA, USA) with a 535 nm excitation filter and a 590 nm emission filter.

### Cell cycle analysis

HT1080 cells were seeded in 10 cm dishes and were infected with lentivirus-harboring shRNA targeting *CSNK1E *for 48 h. The infected cells were harvested, washed once with phosphate-buffered saline (PBS), and resuspended in 1 ml of ice-cold PBS. We transferred 300 μl of PBS-cell suspension to pre-chilled 15 ml tubes, mixed with 5 ml of ice-cold MeOH, and incubated at -20°C overnight. Fixed cells were rehydrated in PBS for 3 h and then pelleted by centrifugation. Cells were reconstituted in 300 μl of PBS containing 60 μg/ml propidium iodide and 50 μg/ml RNase A. Cell cycle profiles were obtained using a FACScalibur flow cytometer (BD Biosciences, San Jose, CA, USA) and CellQuest software (BD Biosciences).

### Real-time quantitative PCR

Total RNA was extracted using the RNeasy kit (Qiagen, catalog number 74104) as described in the manufacturer's handbook. RNA sample (1 μg) was subject to reverse transcription reaction using TaqMan^® ^Reverse Transcription Reagents (Applied Biosystems, Foster City, CA, USA, catalog number N8080234) according to the manufacturer's instructions. Then, quantitative PCR was carried out using Power SYBR^® ^Green PCR Master Mix (Applied Biosystems, catalog number 4367659) and 7300 Real-Time PCR System (Applied Biosystems). The primer sequences used for quantitative PCR were: PER2_F, 5'-GCAAAATCTGAACACAACCC-3'; PER2_R, 5'-CTTTGTGTGTGTCCACTTTC-3'; CYCLINB1_F, 5'-CTGGCTAAGAATGTAGTCATG-3'; CYCLINB1_R, 5'-GGTAGAGTGCTGATCTTAGC-3'; CYCLINA2_F, 5'-CAGCAGCCTGCAAACTGC-3'; CYCLINA2_R, 5'-GAGGTATGGGTCAGCATC-3'; WEE1_F, 5'-GCATTTATGCCATTAAGCGATC-3'; WEE1_R, 5'-GAGAATGCTGTCCAAGCAC-3'; CYCLIND1_F, 5'-CTTCGTTGCCCTCTGTGC-3'; CYCLIND1_R, 5'-CACCATGGAGGGCGGATTG-3'.

The mRNA level of human acidic ribosomal phosphoprotein P0 was measured using the following primers and used as a reference for quantification: RPLP0 F, 5'-ACGGGTACAAACGAGTCCTG-3'; RPLP0 R, 5'-GCCTTGACCTTTTCAGCAAG-3'.

### Western blotting

#### Monitoring cleavage of PARP1 and caspase-3 upon shRNA treatment

We seeded 2 × 10^6 ^HT1080 cells in 10 cm dishes and treated them with 1 μM staurosporine for 16 h. Virus containing shRNAs targeting *CSNK1E *was used to infect HT1080 cells for 48 h. Both dying cells and live cells in each 10 cm dish were harvested and collected in the same 15 ml tubes by centrifuging cell suspensions at 1,000 rpm for 5 minutes. Cell pellets were washed three times with PBS and cells were lysed in 200 μl of denaturing lysis buffer (50 mM HEPES KOH (pH 7.4), 40 mM NaCl, 2 mM EDTA, 1.5 mM Na_3_VO_4_, 50 mM NaF, 10 mM sodium pyrophosphate, 10 mM sodium β-glycerophosphate, 0.5% Triton X-100, and protease inhibitor tablet (Roche, catalog number 11836170001)). Protein content was quantified using a Bio-Rad protein assay reagent (Bio-Rad, Hercules, CA, USA, catalog number 500-00006). Equal amounts of protein were resolved on SDS-polyacrylamide gels. The electrophoresed proteins were transblotted onto a PVDF membrane, blocked with 5% milk, and incubated with rabbit primary antibodies specific to: PARP1 (Santa Cruz, Santa Cruz, CA, USA, catalog number sc-7150); cleaved caspase-3 (Cell Signaling Technology, Danvers, MA, USA, catalog number 9661) overnight at 4°C. The membrane was then incubated in IRDye 800 goat anti-rabbit antibody (Li-cor Bioscience, Lincoln, NE, USA, catalog number 926-32211) at 1:3,000 dilutions for 45 minutes at room temperature. After washing off the unbound antibodies, membranes were scanned using the Odyssey™ Imaging System (Li-cor Bioscience).

## Abbreviations

CDK, cyclin-dependent kinase; CK1ε, casein kinase 1-epsilon; DMEM, Dulbecco's modified Eagle's medium; GI, growth inhibition; hTERT, catalytic subunit of human telomerase; LT, SV40 large T oncoprotein; PARP1, poly(ADP-ribose)polymerase-1; PBS, phosphate-buffered saline; RISC, RNA-induced silencing complex; RNAi, RNA-interference; *shCSNK1E*, shRNA targeting *CSNK1E*; shRNA, short hairpin RNA; ST, SV40 small T oncoprotein; VCM, viral collection media.

## Authors' contributions

WSY and BRS conceived the study, designed the experiments, analyzed the data, and wrote the manuscript. WSY collected the data. BRS supervised the research.

## Additional data files

The following additional data are available. Additional data file 1 displays the growth inhibitory activity of all shRNAs used in this study, statistical analysis, and list of hits. Additional data file 2 is a figure showing the results of testing shRNAs targeting *PER1*, *PER3*, *CRY1*, and *CRY2 *for suppressing IC261-induced growth arrest.

## Supplementary Material

Additional data file 1 Growth inhibitory activity of all shRNAs used in this study, statistical analysis, and list of hits. Click here for file

Additional data file 2 Results of testing shRNAs targeting *PER1*, *PER3*, *CRY1*, and *CRY2 *for suppressing IC261-induced growth arrest. Click here for file
