# Supplementary material for: Inhibition of casein kinase 1-epsilon induces cancer-cell-selective, PERIOD2-dependent growth arrest
Source: Genome Biol. 2008 Jun 2;9(6):R92. doi: 10.1186/gb-2008-9-6-r92 (PMC2481424; doi:10.1186/gb-2008-9-6-r92)
Supplement: Additional data file 2 — Results of testing shRNAs targeting PER1, PER3, CRY1, and CRY2 for suppressing IC261-induced growth arrest. [file gb-2008-9-6-r92-S2.pdf]

Fig. S1

Figure S1. Knocking down other genes of CKI-epsilon subrates did not make cells resistant to IC261 treatment.

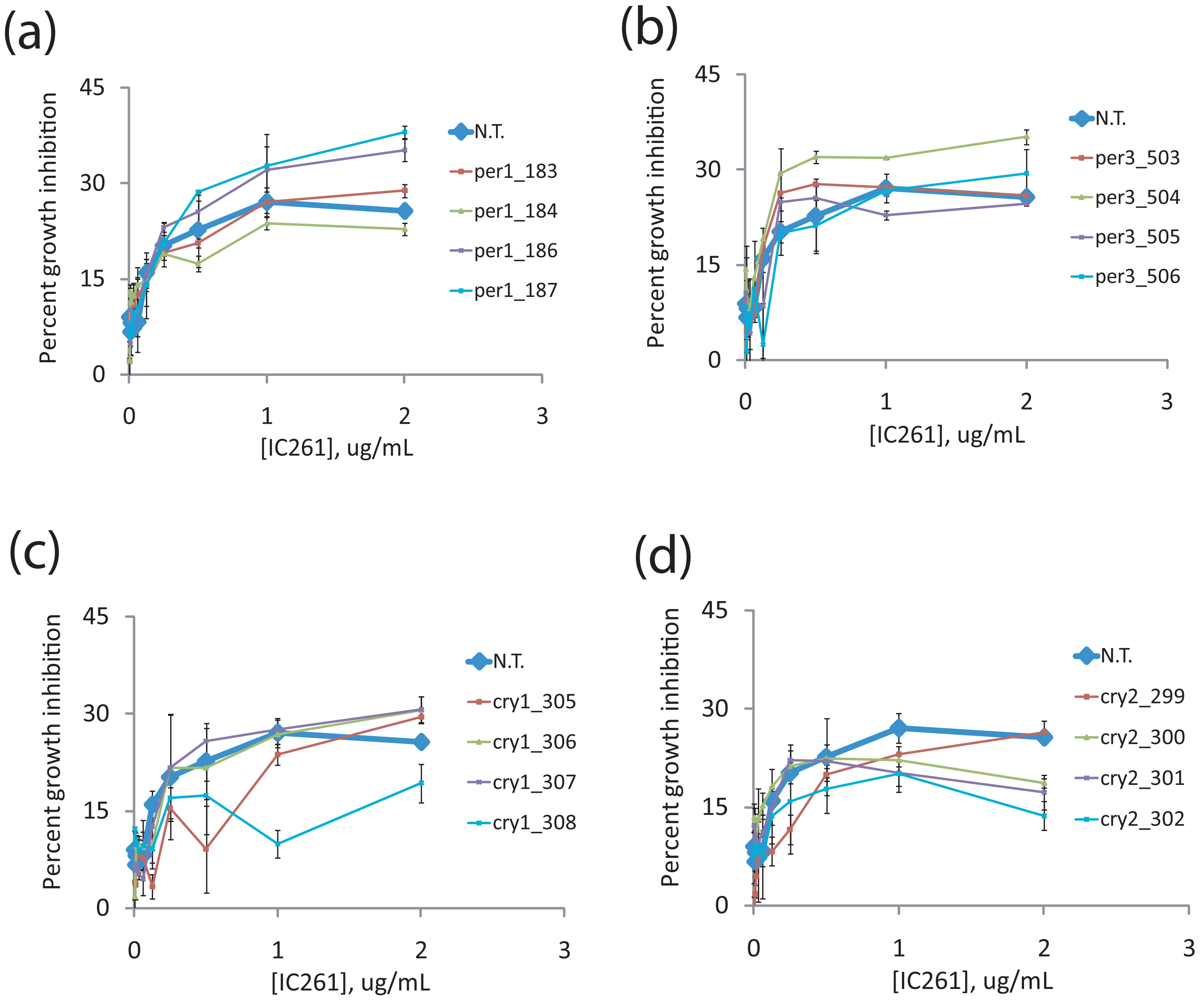

Methods

HT1080 cells were infected with lentiviruses harboring indicated shRNA clones. After two days of incubation, each population of shRNA-expressing cells was treated with indicated concentrations of IC261, a CKI-epsilon kinase inhibitor, for 24 hours. Alamar blue dye was added to the culture to measure cell viability.
